# Temporal integration and decision-making in crocodiles

**DOI:** 10.1242/bio.061844

**Published:** 2025-05-06

**Authors:** Naïs Caron Delbosc, Julie Thévenet, Nathalie Grosjean, Loïc Méès, Nicolas Boyer, Mélanie Schneider, Nicolas Grimault, Nicolas Mathevon

**Affiliations:** ^1^ ENES Bioacoustics Research Lab, CRNL, CNRS, Inserm, University of Saint-Etienne, 42100 Saint-Etienne, France; ^2^ Équipe Cognition Auditive et Psychoacoustique/CRNL, CNRS, Inserm, University Lyon 1, 69500 Bron, France; ^3^ Univ Lyon, CNRS, École Centrale de Lyon, INSA Lyon, University Claude Bernard Lyon 1, LMFA, UMR5509, 69134 Écully, CEDEX, France; ^4^ Institut universitaire de France, 75005 Paris, France; ^5^ École Pratique des Hautes Études, CHArt Lab, PSL University, 75014 Paris, France

**Keywords:** Crocodiles, Sensory perception, Decision-making, Sound, Water surface waves, Temporality

## Abstract

To make appropriate behavioural decisions, animals continuously process a flow of information provided by different sensory channels. Could temporality, i.e. the order in which independent stimuli are perceived, lead the animal to give greater importance to one stimulus than to another? Here we show that the decision of a crocodile to move preferentially towards the source of water surface waves than towards the source of an airborne sound is irrespective of the relative time of arrival of the sound and water vibrations to the animal, as long as the delay between these two stimuli does not exceed a few seconds. To test whether the late arrival of water waves – which travel more slowly than sound – could explain crocodiles' preference for the source of water waves, we controlled the relative timing of stimulus arrival within a time window of a few seconds. Our results reveal that crocodiles preferentially move towards the source of the water waves, whether they arrive after, at the same time as, or before the sound. This suggests that the temporal integration of information from different sensory channels can occur within a certain time window, where the behavioural decision-making remains independent of the arrival order of stimuli. The maximum delay between simultaneously evaluated stimuli probably depends on animal species and context.

## Teaser

**Summary:** Crocodiles' attraction to two sensory stimuli (water vibration and airborne sound) is independent of the order of arrival of these stimuli, within the limit of a certain time window.

## INTRODUCTION

Each animal inhabits its own world, constantly integrating information conveyed by various sensory channels (Partan and Marler, 2002; Von Uexküll, 1992). This diversified information drives the animal's decision-making, leading to a behavioural response adapted to the situation (Higham and Hebets, 2013; Partan and Marler, 1999; Raposo et al., 2012). Although there is obviously considerable diversity between animal species depending on their lifestyle and phylogenetic position, decision-making is frequently based on a combination of multiple pieces of information (Raposo et al., 2012). It may involve some complex cognitive processes, emerging from the probability of an event associated with the information (for example the presence of prey or a predator) and the potential benefit of responding in a particular way (e.g. approach or flee; Petrillo and Rosati, 2021). Importantly, decision-making is a phenomenon with a strong temporal component (Addessi et al., 2011, 2013). It is profoundly influenced by the temporal dynamics of the perception of the various stimuli (order and time of arrival) and by temporal constraints on the behavioural choices to be made (for example, fast approach or rapid retreat depending on the probability of capturing prey or escaping a predator). When an animal perceives a stimulus, it has three main options: ignore it, respond immediately, or wait before responding. If it waits before responding, it may receive other information from other stimuli during this delay. This new information may be complementary, redundant or contradictory to the first stimulus, and is likely to alter the decision that would have been made if the first stimulus had stood alone. Understanding how this successive information is integrated by the receiving animal and modulates its decision-making is therefore fundamental to understanding how an animal navigates its sensory world and responds to the incoming stimuli. In particular, how does the animal manage its decision-making when faced with successive stimuli, where each, if perceived on its own, would have led to a particular and different response? Here, we explore the behavioural response of Nile crocodiles *Crocodylus niloticus* to airborne sound signals and water surface vibrations as a function of the time of arrival at the receiving individual. Indeed, crocodilians are a model of choice: top predators, they spend their time scanning their environment and have to make behavioural decisions according to the stimuli that reach them concomitantly or successively.

Crocodiles are amphibious top predators and are adept at hunting from ambush (Grigg and Kirshner, 2015). Their preferred position is to have their head positioned at the air-water interface, with their nostrils, eyes and ears aligned just above the surface (Di-Poï and Milinkovitch, 2013; Grigg and Kirshner, 2015; Leitch and Catania, 2012). To these classic sensory sensors in vertebrates, crocodiles add a unique system: integumentary sensory organs (ISOs). These mechanoreceptors are extremely sensitive to variations in water pressure. ISOs detect both disturbances at the water surface and displacement under water (Di-Poï and Milinkovitch, 2013; Grap et al., 2015; Leitch and Catania, 2012; Soares, 2002). The distribution of ISOs on the body varies across crocodilian species. Found only on the head in the Alligatoridae, they are present all over the body in the Crocodilidae and Gavialidae, with a particular high density around the jaws (around 9000 ISOs in the Nile crocodile; Leitch and Catania, 2012). Highly sensitive, ISOs enable crocodilians to locate the position of a source of water surface waves (Leitch and Catania, 2012; Soares, 2002) and to distinguish between surface waves with different spectral and temporal properties (Grap et al., 2020).

To assess how crocodiles make decision as a function of the temporal dynamics of stimuli perception, we tested whether the order of arrival of sound and vibration stimuli influences the preference of the receiving animal to move towards the source of one or other of the stimuli. We first compare the propensity of young Nile crocodiles to approach the source of a sound (conspecific contact call) to that of water surface vibrations. By measuring the response of the crocodiles depending on whether the two sources are located in the same place (co-located) or separated, we assess whether there is a possible cumulative effect of the stimuli by examining whether the surface waves, which inevitably arrive after the sound waves if emitted at the same time, could modulate the response to the sound. In the main experiment of our study, we then investigate whether the crocodiles' decision to move towards one or the other stimulus source could depend on the relative time and order of arrival of the two stimuli. Specifically, we test the hypothesis that the decision to move towards the source of the first perceived stimulus is modified if the stimulus arriving later is more attractive. We also test whether the arrival delay between the two stimuli influences this possible change in decision making. To carry out these experiments, we relied on the fact that a wave on the surface of water propagates about a thousand times more slowly than a sound wave in air (about 0.3 m s^−1^ and 340 m s^−1^, respectively; Grap et al., 2020). This enabled us to precisely control the relative arrival times of sound and vibration stimuli at the receiving individual. Overall, these experiments shed light on the sensory preferences and cognitive mechanisms underlying decision-making in crocodilians.

## RESULTS

### Experiment 1: both water surface waves and airborne sounds are independently attractive to crocodiles

#### Experiment 1a: separated sources

**Fig. 1.**
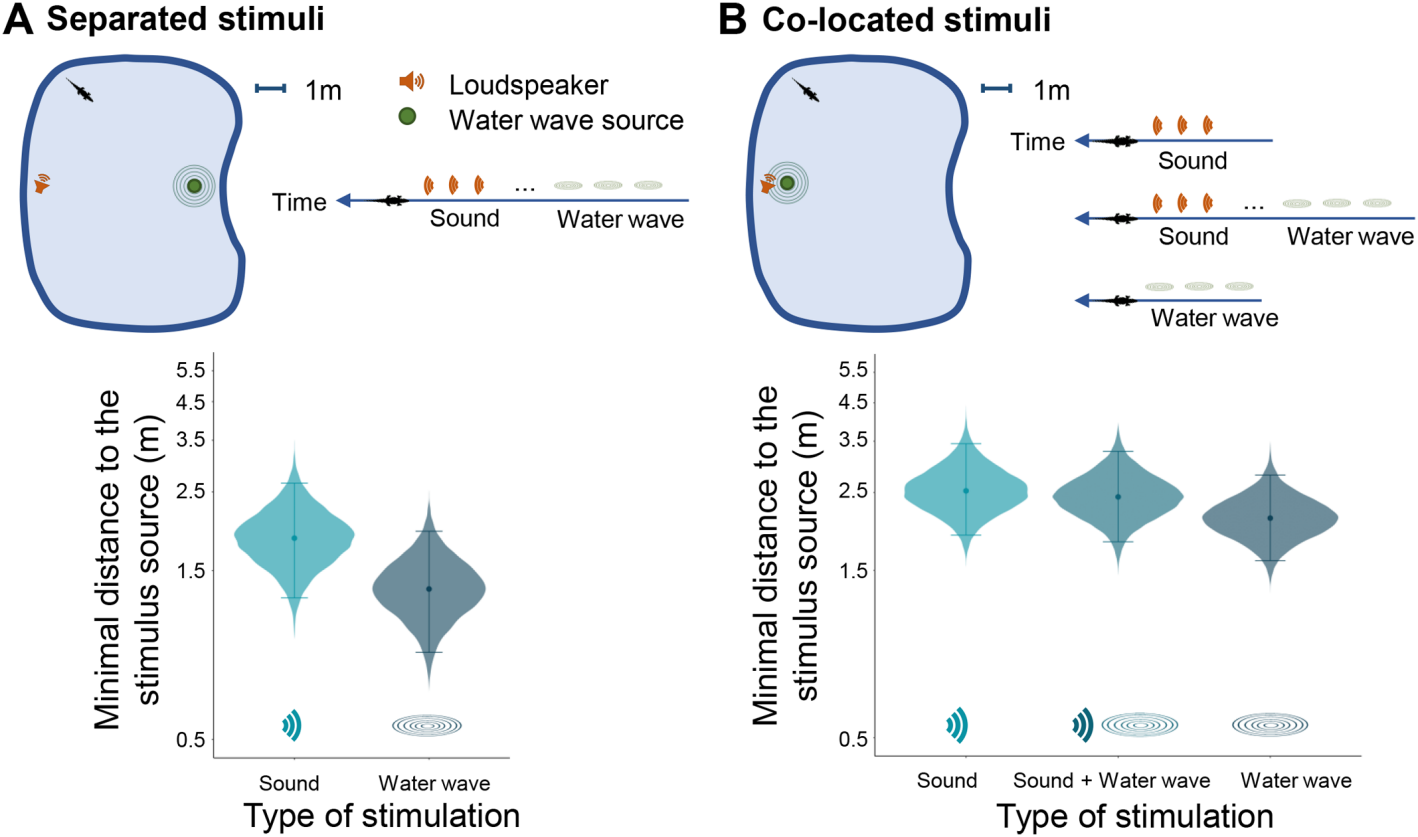
**An airborne sound and a water surface wave independently attract young crocodiles.** (A) Experiment 1a. An airborne sound and a water surface wave are emitted at the same time from two different locations (separated stimuli; 22 trials). The crocodiles (*N*=9 individuals) were attracted to both stimuli, with a tendency to approach more the vibration source. (B) Experiment 1b. Sound (22 trials) and water surface waves (27 trials) are emitted alone or altogether from the same location (co-located stimuli; 22 trials). Crocodiles approached the stimulus source whatever the stimulus. There is no interactive effect between sound and vibration when they are presented together. Violin plots represent the shortest distance to the stimulus source reached by the crocodile (fitted value of the median of the posterior distribution with 95% CI). The distributions are derived from repeated observations across individuals.

In this experiment, the two stimuli – auditory and vibratory – were emitted from two different locations in the experimental basin (‘Separated condition’, Fig. 1A). Comparison of the minimum approach distances to the stimulus source shows a slight preference of the crocodile for the vibration source [Fig. 1A; difference between approach distance in response to vibration and sound stimuli: −50 cm, 95% CI (−126, 16), 93.76% of the posterior distribution is negative, BF=0.95; Table S2]. As the delay between the arrival of the sound and that of the water vibrations depended on the position of the crocodile relative to the emitting sources (median delay=−7.7 s; Fig. S1A), we then examined the potential effect of this delay on the crocodiles' response. To do this, we divided our data into two subsets: on the one hand, trials for which the arrival delay between sound and vibration was longer than the median delay (i.e. >7.7 s), and on the other, trials for which this delay was shorter (<7.7 s). We found that crocodiles were preferentially attracted to the vibration source when the vibrations arrived with a delay of less than 7.7 s after the sound [Fig. S1B; difference between approach distance in response to vibration and sound stimuli: −23 cm, 95% CI (−157, 121), 66.09% of the posterior distribution is negative, BF=0.44 for delay >7.7 s; −57 cm, 95% CI (−200, 60), 84.52% of the posterior distribution is negative, BF=1.19 for delay <7.7 s].

**Fig. 2.**
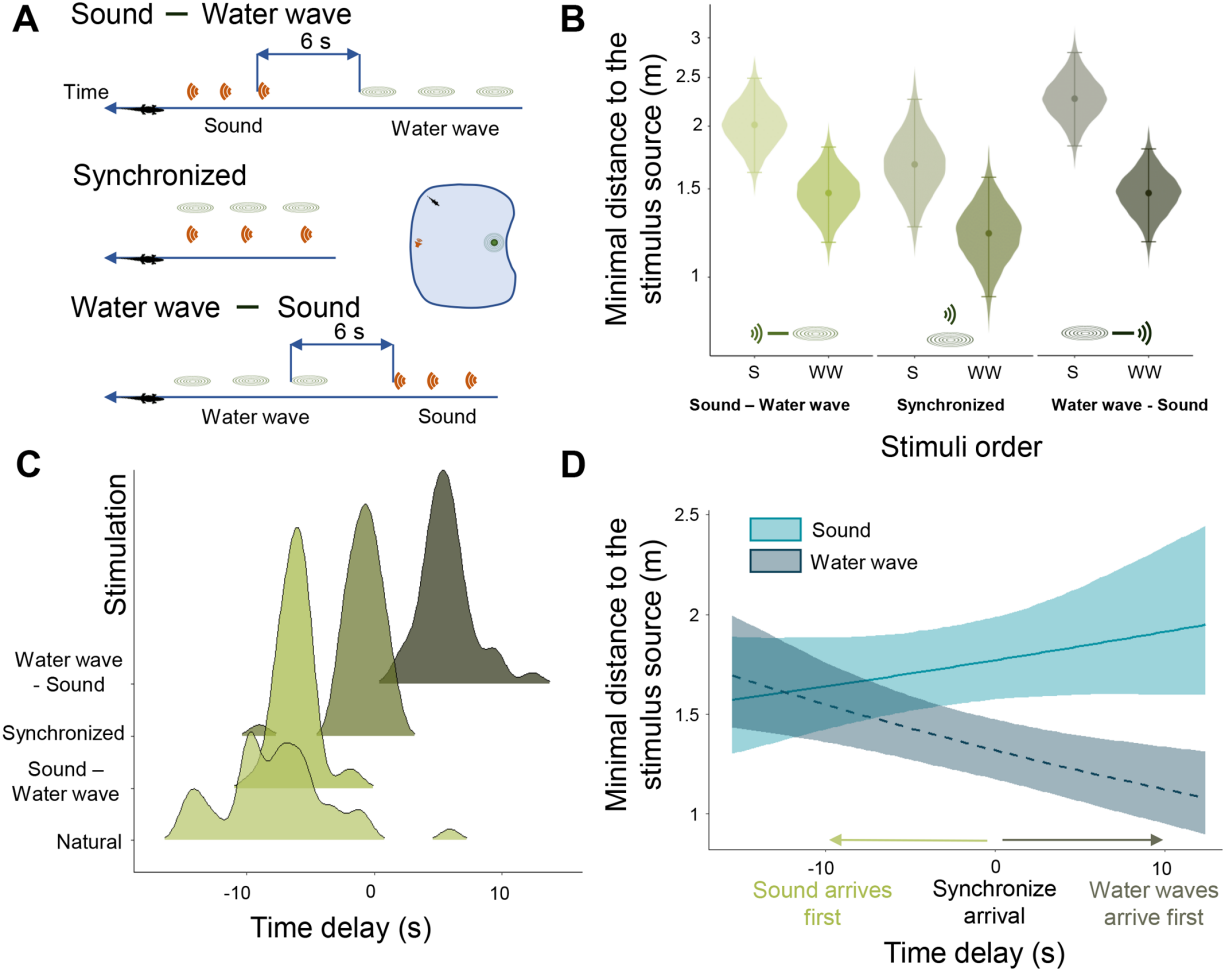
**Effect of stimuli order and arrival time on the crocodile response.** (A) Experiment 2. To control the order of arrival of stimuli at the crocodile, sound (S) and water surface waves (WW) are emitted from two different locations with different delays (40 trials for each condition). In the ‘sound-vibration’ condition, the onset-to-onset delay between the last sound and the first water wave is 6 s. In the ‘synchronized’ condition, the onset-to-onset delay between the first sound and the first water wave is set to zero. In the ‘vibration-sound’ condition, the onset-to-onset delay between the last water wave and the first sound is 6 s. (B) Regardless of the order of arrival of the two stimuli, the crocodiles (*N*=10 individuals) preferentially approached the water surface wave source. Violin plots represent the minimum distance to the stimulus source reached by the crocodile (fitted value of the median of the posterior distribution with 95% CI). (C) Distributions of measured time delays between the arrival of the vibration stimulus and the arrival of the sound stimulus at the crocodile depending on the playback condition (‘natural’ condition=the two stimuli are emitted at the same time). (D) The later the sound arrives, the more the crocodiles are attracted to the source of the vibration [Bayes Factor on the interaction between the delay and the type of stimulation=19,100; slope for vibration=−2 cm, 95% CI (−4, −1), 99.88% of posterior distribution is negative; slope for sound=1 cm, 95% CI (−1, 4), 90.45% of posterior distribution is positive]. The distributions are derived from repeated observations across individuals.

#### Experiment 1b: co-located sources

In this experiment, the two stimuli – auditory and vibratory – were emitted from the same position in the experimental tank, either together or alone (Fig. 1B). Crocodiles tend to be equally attracted to the stimulus source whether the sound and the vibration were emitted separately or at the same time [Table S2; difference in minimum approach distance to the source for sound and vibration emitted separately: −41 cm, 95% CI (−120, 26), 88.99% of the posterior distribution is negative, BF=0.48; difference in minimum approach distance between vibration emitted alone and sound and vibration emitted at the same time: −31 cm, 95% CI (−107, 38), 82.68% of the posterior distribution is negative, BF=0.329]. For the sound alone condition, the response delay was 71.5±82.3 s.

Overall, based on the results from experiment 1, we cannot conclude that either sound or vibration are more attractive for crocodiles than the other. Moreover, when the delay between the arrival of the sound wave and that of the vibration is sufficiently short (less than 7.7 s in our experimental conditions), the preference of crocodiles for the vibration source tended to increase. In experiment 2, below, we tested whether this potential preference is dependent on the order and arrival time of the stimuli.

### Experiment 2: the order of arrival of stimuli does not affect their respective attractivity

The aim of this second experiment was to test whether the temporality of the two stimuli modulates their respective attractivity. As detailed in the Materials and Methods section, we tested crocodiles (*N*=10) in three configurations, where sound and vibration arrived at the crocodile either at the same time (‘synchronized’), or delayed (sound then vibration, or vibration then sound; with a delay of around 6 s between the two stimuli; Fig. 2A; see also Fig. 2C for the exact distribution of temporal delays measured from the videos). We also re-tested the same configuration as in experiment 1a (‘natural condition’), where the sound and the vibration were emitted at the same time and the time lag between the two stimuli varied according to the crocodile's position relative to the two sources (Fig. 2C).

As shown in Fig. 2B, whether the two stimuli were perceived at the same time, or 6 s apart did not alter the crocodile's behavioural response. In all cases, the crocodiles significantly prefered to approach the source of the vibrations, whether they were perceived before, at the same time, or after the sound [water wave, sound: −79 cm, 95% CI (−126, −42), 99. 99% of the posterior distribution is negative, BF=100.79; synchronized: −45 cm, 95% CI (−100, −3), 98.38% of the posterior distribution is positive, BF=17.68; sound, water wave: −54 cm, 95% CI (−98, −16), 99.70% of the posterior distribution is negative, BF=8.65;  Table S3].

To complete this analysis, we measured the exact delay between the arrival of the sound stimulus and the vibratory stimulus to the crocodile as a function of the animal's position in the experimental tank. The distribution of these delays is shown in Fig. 2C. As illustrated in Fig. 2D, the crocodiles' preference for the vibration source increased with the later arrival of the sound. The order of the stimuli is irrelevant: in the time window considered, the sound can arrive before, at the same time as or after the vibration.

## DISCUSSION

Here we show that when crocodiles preferentially move towards a source of water vibrations rather than a source of airborne sound, their preference for the vibration source remains unchanged whether the vibrations reach the animal after, at the same time as or before the sound, as long as the delay between the two stimuli does not exceed several seconds. Thus, instead of being sequential – and the first or last stimulus perceived wins the animal's preference – this assessment of the relative relevance of two stimuli and the subsequent decision-making take place within a certain time window. This result demonstrates that an animal can decide, independently of the respective moment at which two different stimuli are perceived, which one it will prefer over the other.

While previous studies have demonstrated that crocodiles are highly sensitive to water surface vibrations (Di-Poï and Milinkovitch, 2013; Grap et al., 2015; Leitch and Catania, 2012; Soares, 2002), and that they accurately locate a source of sound (Bierman and Carr, 2015; Papet et al., 2020), we did not yet have strong evidence that they are also able to locate the position of a source of water surface vibration. For example, it had been observed that an alligator orients itself towards the point of impact of drops falling into the water, but the distance between the individual and the impact was only a few tens of centimetres. It cannot be ruled out that the alligator responded to the sound of the impact rather than the vibration of the water (Soares, 2002). A similar observation was made with a Nile crocodile and an alligator (*Alligator mississippiensis*), where the individuals approached the impact of food pellets on the surface of the water. In this instance, olfactory cues potentially offered localization information in addition to vibrations (Leitch and Catania, 2012). The experiments reported in our study suggest that a crocodile is capable of locating the position of a source of low-amplitude vibrations propagating on the water surface, even if that source is several meters away (see https://zenodo.org/records/10401637). Measuring the accuracy with which crocodiles locate a source of vibration will, however, require further experimentation. This ability to locate the position of a source of water vibration certainly relies on the ISO mechanoreceptors, whose role in vibration perception has been widely demonstrated (Di-Poï and Milinkovitch, 2013; Grap et al., 2020, 2015; Leitch and Catania, 2012; Soares, 2002). As ISOs are widely distributed throughout the animal's body (Leitch and Catania, 2012), it is likely that mechanisms similar to those used by the auditory system to localize a sound source are involved in the localization of a water vibration source (i.e. based on the comparison of stimulation of different regions of the body, in a manner analogous to the interaural time and intensity differences in hearing). In addition, the dispersion of water surface waves as they propagate could provide distance cues to the crocodile based on the frequency content of the waves reaching it (see the Materials and Methods section for details).

The most original aspect of our work was to demonstrate that a crocodile can decide to respond to consecutive stimuli independently of their order of arrival. In other words, this animal integrates the information from the two stimuli and makes the decision to move towards one or other of the stimulus sources over a certain time window. The maximum duration of this decision-making window appears to be less than 7-10 s in our experimental context. Within this time window, the order of arrival of the stimuli is of little importance: the crocodile has a preference for responding to one stimulus (the vibration) rather than another (the sound) and shows this preference whether the preferred stimulus arrives before, during or after the less attractive stimulus. This observation, supported here experimentally, suggests that the behavioural response to the information carried by each of the two competing stimuli is not a simple reflex but involves cognitive processes involving working memory. The information (in terms of modality and direction of source) provided by the stimulus that arrives first has to be stored in the animal's working memory. The decision to go towards the source of the stimulus is thus delayed and will eventually be modulated by the perception of the second stimulus. This working memory seems to fade after about 10 s, as the preference for approaching the source of vibration over the source of sound decreases when the sound arrives more than 7 s before the vibration (Fig. S1B and Fig. 2D).

In our experiment, the sound and vibration stimuli do not represent the components of multimodal information. The information is not redundant between the two modalities, which explains why neither reinforces the response to the first. Sound and vibration are therefore perceived independently and do not seem to be considered as linked by the crocodile. This sensory configuration is therefore different from that observed in a previous study in which a food odour modified the behavioural response of Nile crocodiles to sound stimuli (Chabrolles et al., 2017).

Our study is obviously not free from elements that may complicate the interpretation of the results. One point is that the initial distances between the crocodile and the source of sound and vibration were not equal. On average, the crocodiles were closer to the source of vibration: 3.5 m compared to 4.4 m for experiment 2. It cannot be ruled out that this greater proximity may have influenced the crocodiles' preference for the vibration source but *a priori* there is no acoustic cue to determine the distance at which sounds are emitted (unlike water surface waves). However, this does not undermine the fact that the order in which the stimuli reached the crocodile did not influence their decisions. Indeed, the parameter we took into account was the relative arrival time between the two stimuli. This relative time was precisely measured, taking into account the relative distances of the crocodile from each of the two sources. The second factor is that the two stimuli – sound and vibration – are of a different physical nature. Consequently, characteristics such as signal intensity (amplitude) or others (spectrum, directivity, phase) cannot be used to assess the relative position of the two sources, and only the relative arrival times and order matter.

Our study documents the temporal trade-off accompanying decision-making in a crocodile. In previous pilot experiments, we observed a crocodile deciding to move towards a loudspeaker about 10 min after the end of the sound stimulus (Grimault & Mathevon, personal observation). It is probable that crocodiles can strongly delay their behavioural response to a stimulus, which suggests memory capacities that have yet to be explored. The context reported here already testifies to the robustness of their decision-making mechanism. The arrival of a second stimulus does not alter the decision to move towards the source of the first stimulus. This persistence in the face of competing stimuli suggests advanced cognitive processing. Furthermore, our experiments show that crocodiles are capable of integrating information from two distinct sensory modalities over time. This ability is well suited to their lifestyle as ambush predators, integrating complex and successive environmental signals and making decisions over large time windows. In fact, as far as we know, this discovery of a time window for decision-making in the crocodile is unprecedented in any animal. Having to decide how to behave in the face of competing stimuli arriving at different times is probably a common situation in the daily lives of most animals.

It would now be useful to determine the factors likely to modify the duration of the temporal window during which stimuli of different natures are integrated before the animal responds. It is likely that the duration of this window depends on the animal species. It is also likely to depend on the information conveyed by the stimuli, with some information requiring an immediate response from the receiving animal (e.g. the perception of the presence of a predator). It is likely that the crocodile, with its habit of hunting from ambush, its heterothermy that makes it more sparing of its movements, and its position as a top predator, decides to react behaviourally after taking the time to assess the situation over a relatively long time window.

## MATERIALS AND METHODS

### Location and tested animals

**Fig. 3.**
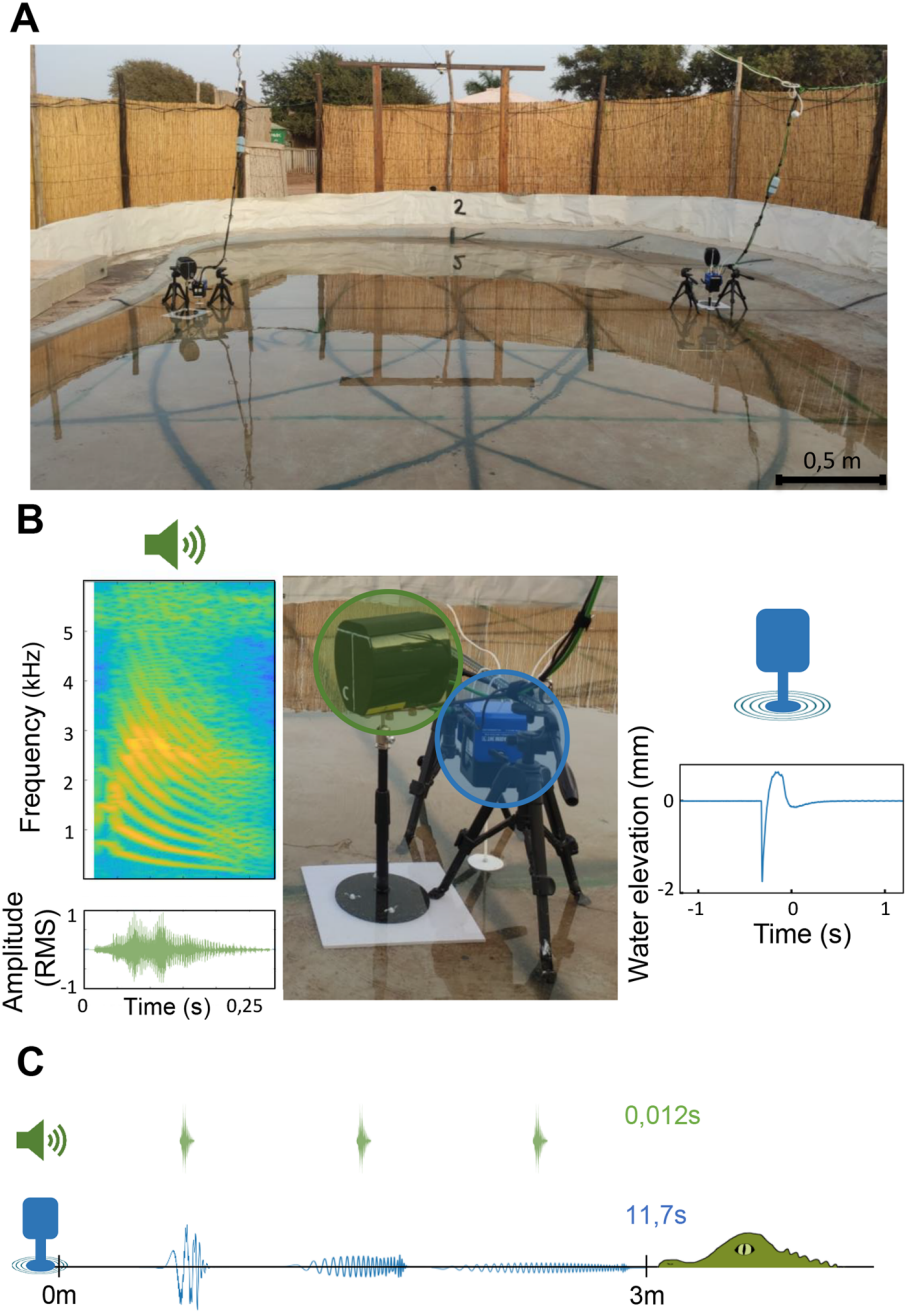
**Experimental set-up and sensory stimuli.** (A) Experimental basin with the sound and vibration emitting devices (remote-controlled loudspeakers and vibrating devices). The black lines on the bottom of the basin are drawn at distances of 2, 3, and 4 m from each device allowing us to determine the initial position of the crocodile before sending the stimuli. (B) From left to right: spectrogram and oscillogram of a crocodile call; the duo loudspeaker (green)/vibratory device (blue); surface wave created by the vibratory device at the source of stimulation. (C) Airborne sounds propagate more rapidly than water surface waves and are less attenuated (wave drawings are not at scale).

We carried out the experiments at the ‘Crocoparc’ zoo (Agadir, Morocco). All experiments were performed in accordance with relevant guidelines and regulations in Morocco and under the supervision of zoo's staff. We worked with 19 naïve juvenile Nile crocodiles *C. niloticus* (one individual tested per night across two experimental sessions). The animals were kept outside, in an enclosure not accessible to the public. The experiments were conducted during the night in a dedicated basin (6 m×8 m; Fig. 3A). Each crocodile was tested separately using two pairs of loudspeaker/vibratory device.

### Experimental signals

We used two types of stimuli: an airborne sound and a vibratory stimulus (vibration of the water surface). The sound was a contact call from a young Nile crocodile. To limit habituation, we constructed 14 different sequences, each containing three identical contact calls from the ENES data bank (recorded in the Okavango Delta, Botswana, by T. Aubin and N. Mathevon; Fig. 3B). Contact calls are known to maintain cohesion in groups of young crocodilians and are therefore naturally attractive to them (Vergne et al., 2011). The average duration of a call was 0.27±0.04 s and the average duration of silence between calls was 3.38±0.12 s, giving sequences that lasted an average of 7.52±0.12 s. The sound level of these calls was 68 dB (C weight as a flat approximation, slow time weighting, at 1 m from the source). We used FoxPro Fusion loudspeakers (Lewistown, Pennsylvania, USA) for experiment 1 and Audio Pro - Bravo Allroom Sat (Helsingborg, Sweden) for experiment 2 (placed approximately 10 cm above the water surface).

The vibratory stimulus was a series of three water surface waves created by a 2.5 cm radius disc striking the water surface, with a delay of 7.5 s between the first and last impact on the water surface (vibrating pot PCB Piezotronics - K2004E01; Depew, New York, USA). The amplitude of the surface wave decreases rapidly with propagation distance (see Fig. 3C). The wave amplitude, as received by crocodiles, depends on their distance from the source of stimulus and was estimated from a calibration in a large tank containing water at rest. Surface height over time was measured at different distances from the stimulation source (75, 300 and 600 mm) using a confocal chromatic sensor (µ-epsilon - IFS 2400-10; Ortenburg, Germany), at 1 kHz with 0.4 µm resolution. The wave amplitude was deduced from the time signal as the maximum peak-to-peak variation. The decay of wave amplitude as a function of propagation distance is described by a best-fit curve of the form 
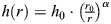
, where *r*_0_ is the disk radius, and where the water height *h*_0_ at the disk boundary and decay coefficient α are found to be *h_0_*=1.215 mm and α=1.14 (see the supplementary material for details).

Wave propagation at the water surface is subject to dispersion. In other words, waves of different frequencies travel at different speeds. The surface wave stimulus used in this study was generated by a circular disk striking the water surface. This signal is broadband and can be considered as a superposition of numerous sinusoidal waves. As each component of this spectrum travels at different speeds, the shape of the signal changes as it propagates (see Fig. 3C). Over long distances, the signal received by the crocodile has a variable frequency content, ranging from low to high frequencies (see supplementary materials). At such distances, we were unable to measure the water displacement under the water surface. We assume that this displacement can be considered as negligible.

### Experiment 1 – experimental conditions and playback protocol

We conducted this experiment in October 2021, with young captive naïve Nile crocodiles (*N*=9 individuals; size: 50.8±8.7 cm; sex unknown). The average air temperature was 21.4±1.8°C and the average water temperature was 26.1±2.6°C. With this experiment, we wanted to determine whether a crocodile was attracted by both stimuli, auditory or vibratory, whether it had a certain preference for one of the two stimuli, and whether the combination of these two stimuli was likely to increase the crocodile's approach towards the source of the stimuli. A different crocodile was tested each night. Before each experimental session, the crocodile to be tested was placed in the experimental basin, where two loudspeaker/vibratory device duos had been previously positioned (Fig. 3A). To allow the crocodile to acclimatise to the change of environment between the enclosure where it was kept with its conspecifics and the experimental basin, each individual was transferred to the experimental basin at least 1.5 h before the start of the experiment.

Two different conditions were tested: separated and co-located stimuli (Fig. 1). In the separated condition (experiment 1a), the sound and vibration stimuli were emitted from two different locations at the same time (Fig. 1A; 22 trials). As a consequence of difference in propagation speed between sound and vibration, the crocodile perceives the sound first, then the water surface wave. In the co-located condition (experiment 1b), the stimuli were sent from the same location, either alone (‘sound’ condition, 22 trials; ‘water wave’ condition, 27 trials, Fig. 1B), or together (‘sound+water wave’ condition, 22 trials, Fig. 1B). The location of the loudspeaker/vibratory devices in the basin were randomly changed between each crocodile.

The mean initial distance between the crocodile and the sound source was 5.07±1.24 m. The mean intensity level of sound was 62 dB at 1 m (minimum: 60 dB, maximum: 65 dB; see Thévenet et al., 2022 for details on sound intensity measurements and propagation). For the water wave source, the mean initial distance between the crocodile and the source was 4.58±1.44 m (see Table 1 for the corresponding height, i.e. peak to peak, of the water wave).

**Table 1.** Attenuation of water surface waves with distance

Experiment 1	Initial distance (m)	Mean (s.d.)	Minimum	Maximum
		4.58 (1.44)	1.32	8.00
	Height of water wave, peak to peak (*μm*)	At mean distance	At minimum distance	At maximum distance
		6.4	26.3	3.4
Experiment 2	Initial distance (m)	Mean (s.d.)	Minimum	Maximum
		3.51 (0.94)	0.60	5.80
	Height of water wave, peak to peak (*μm*)	At mean distance	At minimum distance	At maximum distance
		8.7	64.6	4.9

The height of the water wave reaching the crocodile depends on the distance from the source.

Each crocodile received randomly two to five renditions of each condition with each repetition spaced at least 10 min apart. We always waited for the crocodile to become stationary as it was free to move around the basin. For each trial, we quantified the crocodile's approach by measuring its minimum distance from the source of the stimulus reached during the 5 min post-stimulation observation period. This observation time may seem unusually long for measuring an animal's response to a stimulus. However, it is appropriate in the case of our model. Indeed, young crocodiles can respond after a fairly long latency period (on average 67±88 s in the present experiments). We have modelled this minimum distance with a Bayesian model applied to the logarithm of the variable (see statistical analysis below).

### Experiment 2 – experimental conditions and playback protocol

We conducted this experiment in July 2022, with young captive naïve Nile crocodiles, (*N*=10 individuals; average total size was 66.5±5.7 cm; sex unknown). The average air temperature was 25.3±3.9°C (+18% compared to 2021) and the average water temperature was 28.3±1.9°C (+8% compared to 2021). Given that it was not possible to identify the individual identity of the tested crocodiles from one year to the next, it is possible that some individuals tested in 2022 were part of the cohort tested in 2021.

In this second experiment, we used the same stimuli as in experiment 1. Four conditions were tested. In all cases, the auditory and vibratory stimuli were emitted from two different locations. The first, so-called ‘natural’ condition involved playing both stimuli simultaneously (as in the separated condition of experiment 1a). Because of the different propagation speed, the surface waves arrived at the crocodile after the sound, the length of the delay between the arrival of the two stimuli being dependent on the crocodile's position relative to the sound and vibration sources (Fig. 2C). In the other three conditions, we controlled the duration of the delay between the arrival of the two types of waves at the crocodile. In the sound-water wave condition, the onset-to-onset delay between the last sound and the first water wave was set at 6 s. In the synchronized condition, the onset-to-onset delay between the first sound and the first water wave was set to zero. In the water wave-sound condition, the onset-to-onset delay between the last water wave and the first sound was 6 s (Fig. 2A).

To accurately control these delays between the arrival of the two stimuli, we employed the following method. Circular arcs with radii of 2 m, 3 m, and 4 m, centred on each speaker/vibratory device pair, were traced on the pool floor, allowing us to estimate the approximate position of the crocodile in relation to the source of the stimuli (see Fig. 3A for a photograph of the experimental set-up). Prior to the experiments, we had also prepared combinations of sound/vibration stimuli on the computer controlling the stimuli emission, setting delays corresponding to the different positions of the crocodile. At the time of each trial, we decided which combination to play according to the desired delay and the crocodile's position relative to the sender sources. This method enabled us to effectively control the order and timing of stimuli arriving at the crocodile, whatever its position in the basin. Obviously, it was not possible to predict combinations of stimuli corresponding to all possible positions of the crocodile in the tank, and we used a predefined set of combinations corresponding to distances ranging from 2 m to 4 m with intervals of 0.5 m. When analysing the recorded videos, we measured the exact position of the crocodiles and calculated the exact delay. This resulted in a distribution of data around each delay value (Fig. 2C). In this experiment, the location of speaker/vibratory device in the basin was kept identical for all crocodiles and all trials.

As in the first experiment, the crocodile was placed in the experimental basin for at least 1.5 h before testing began. The different conditions were presented to the crocodile in random order (four trials for each temporal condition and each crocodile). Auditory and vibratory stimuli were emitted randomly by the different sources. On average, the initial distance between the crocodile and the sound source was 4.45±1.01 m and the initial distance between the crocodile and the vibration source was 3.51±0.94 m (see Table 1 for the corresponding peak-to-peak water wave height). The average sound intensity level was 62 dB at 1 m (minimum: 60 dB, maximum: 68 dB). Two tests were separated by a minimum period of 10 min, and we always waited for the crocodile to become stationary.

When the disc of the vibrating device hits the water surface to generate the surface wave, it produces a low-intensity sound. To test whether the crocodiles were sensitive to this sound, this second experiment also included a control condition in which this sound was reproduced from a loudspeaker. The crocodiles paid almost no attention to this sound: the minimum approach distance to the loudspeaker was significantly higher [+48 cm, 95% CI (14, 110), 99.81% of the posterior distribution is positive, BF=6.85; Table S1].

### Analysis of behavioural reaction to stimuli

For all trials, the behaviour of the tested crocodiles was observed and filmed by four infrared cameras (ABUS TVCC34010; Wetter, Germany). We analysed the recorded videos using Kinovea video analysis software (Charmant and contributors, 2021). Before processing the videos, we took care to correct for camera lens distortion and perspective error. We characterized the crocodile responses to stimuli by measuring distances travelled by the crocodile as a function of stimulus source positions.

### Statistical analysis

All statistical analyses were performed in R (v.4.1.2) using Bayesian statistics. Bayesian statistics offer a broader and more adaptable framework than the frequentist approach. One key advantage of Bayesian statistics is its ability to handle complex models and datasets. Bayesian methods can accommodate a wide range of models, even when sample size is limited. Another strength of Bayesian statistics lies in its ability to quantify uncertainty in a more intuitive and meaningful way (Kruschke and Liddell, 2018). By generating posterior probability distributions, Bayesian analysis provides a comprehensive characterization of uncertainty and allows to estimate the size of an effect (Kruschke and Liddell, 2018; Van De Schoot and Depaoli, 2014; Van De Schoot et al., 2017).

The minimum approach distance to the stimulus source was modelled using Bayesian mixed models, fitted in the Stan computational framework (brms package; Bürkner, 2017). For all models, the identity of the tested crocodile was considered as random factor. We modelled the effect of stimulation on the behavioural response. All models were based on four chains of 10,000 iterations with 5000 warm-up samples. We used mildly informative priors, which can be used for small sample sizes (Van De Schoot et al., 2017). Specifically, as we did not know the direction (positive or negative) of the effect of stimuli, we chose priors with a normal distribution centred at 0: N(0, 2.5) for the effect on the minimal (Van De Schoot and Depaoli, 2014). The quality of the models was assessed using the Gelman-Rubin criterion (Rhat=1; Gelman and Rubin, 1992) and by visual checking. Fitted values and contrasts were reported using the median of posterior distributions and 95% credible intervals, reported in the text as 95% CI. This interval means that there is a 95% probability that the regression coefficient resides between the upper and lower limit and indicates the 95% most probable values of the parameter, given the data (Kruschke and Liddell, 2018; Makowski et al., 2019; Van De Schoot and Depaoli, 2014; Van De Schoot et al., 2017). The posterior distribution ranges from 0 to 100% and correlates strongly with the frequentist *P*-value, meaning that *P*-values of 0.1, 0.05, 0.01 and 0.001 correspond approximately to a proportion of the posterior distribution that is positive (or negative) of 95%, 97.5%, 99.5% and 99.95%, respectively (Makowski et al., 2019). We also calculated Bayes factors (BF) that provides direct measures of the relative support that data provide to the competing hypothesis (Van De Schoot et al., 2017; Dienes, 2011; Makowski et al., 2019). BFs represent a continuous measure of evidence: BF>3 is considered as data supporting moderate evidence for H1, BF>10 is considered as strong evidence for H1, BF<1/3 is considered as moderate evidence for H0, and BF<1/10 is considered as strong evidence for H0. BFs between 1/3 and 3 indicate that there is insufficient evidence to draw any conclusion for or against either hypothesis (Dienes, 2011, 2014; Keysers et al., 2020).

### Calibration of the surface waves amplitude decay with propagation distance

The waves generated in the experiment and propagating at water surface are almost circular. Their amplitude decreases fast with propagation. To quantify this waves amplitude decay, measurements was carried out in a tank containing water at rest, for different water depth *H* and two different disk radii (*r*_0_=25 mm and *r*_0_=15 mm). Surface height *h* was measured as a function of time at different distances from the stimulation source (*r*=75, 300 and 600 mm, by means of a confocal chromatic sensor (μ-epsilon - IFS 2400-10; Ortenburg, Germany), at 1 kHz with 0.4 μm resolution. The actual movement of the sticking disk generating the initial water surface perturbation is also measured, in air.

**Fig. 4.**
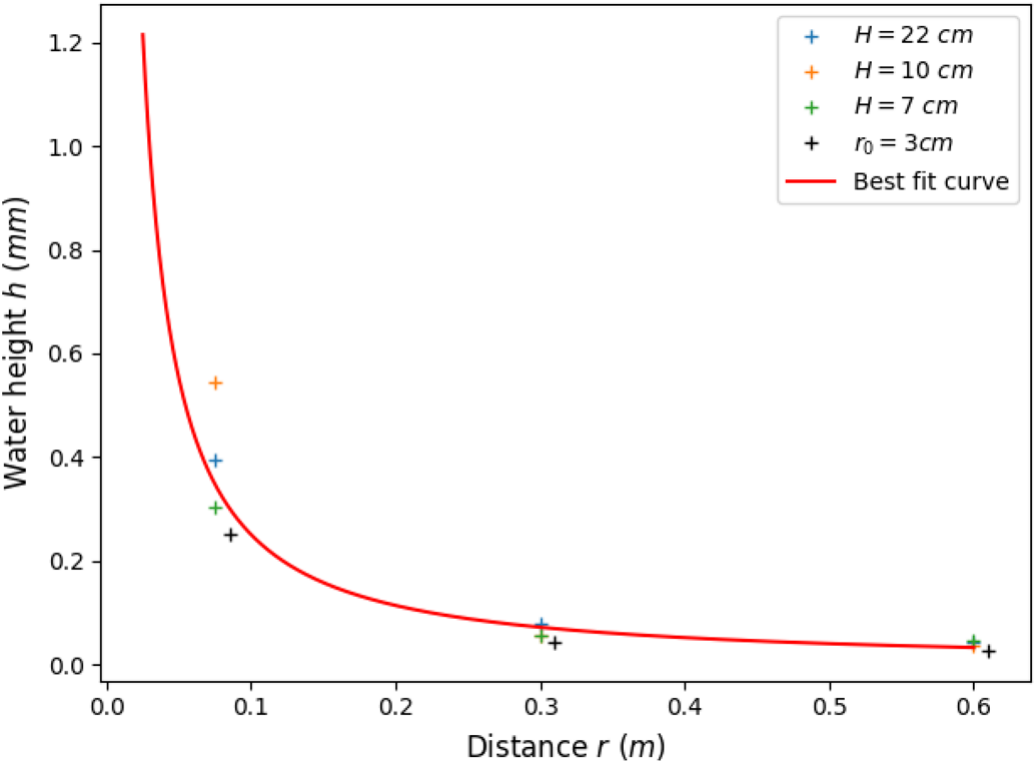
**Calibration curve.** Water height *h* as a function of the propagation distance *r* and calibration curve that best fit the whole data set. Measurements were done for various water depth *H*=22, 10 and 7 cm and two different striking disk radii (*r*_0_=25 mm and *r*_0_=15 mm).

The shape of the water wave changes with propagation, due to attenuation and dispersion effects. After a few centimetres, it has the form of a regular oscillation, limited by a variable envelope (see the supplementary material). The amplitude of the envelope decreases with propagation distance while its duration increases. The wave amplitude was thus defined from the time signal as the maximum peak to peak variation. The measurements results are summarised in Fig. 4 where averaged results obtained in the different conditions are plotted together with a best fit curve. The plots clearly show variation from one case to another. These variations are not due to an effect of water depth *H* (nor the radius *r*_0_) but to the imperfect repeatability of the experiments. They are observed whenever the disk is reset on the surface water. In consequence, best fit parameters are determined from the whole data set, to account for these variations. The fitting curve of Fig. 4 as the form 
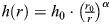
 where *r*_0_ is the disk radius, *h*_0_ is the water height at the disk boundary and *α* is a parameter close to unity that describe the amplitude decay, in a form close to the 1/*r* decay of a circular wave. The unknown coefficients *h*_0_ and *α* providing the best fit are found to be *h*_0_=1.215 mm and *α*=1.14. The best fit value of *h*_0_ is very close to the one extracted from the striking disk movement (in air), which is 1.195 mm.

### Surface waves propagation

**Fig. 5.**
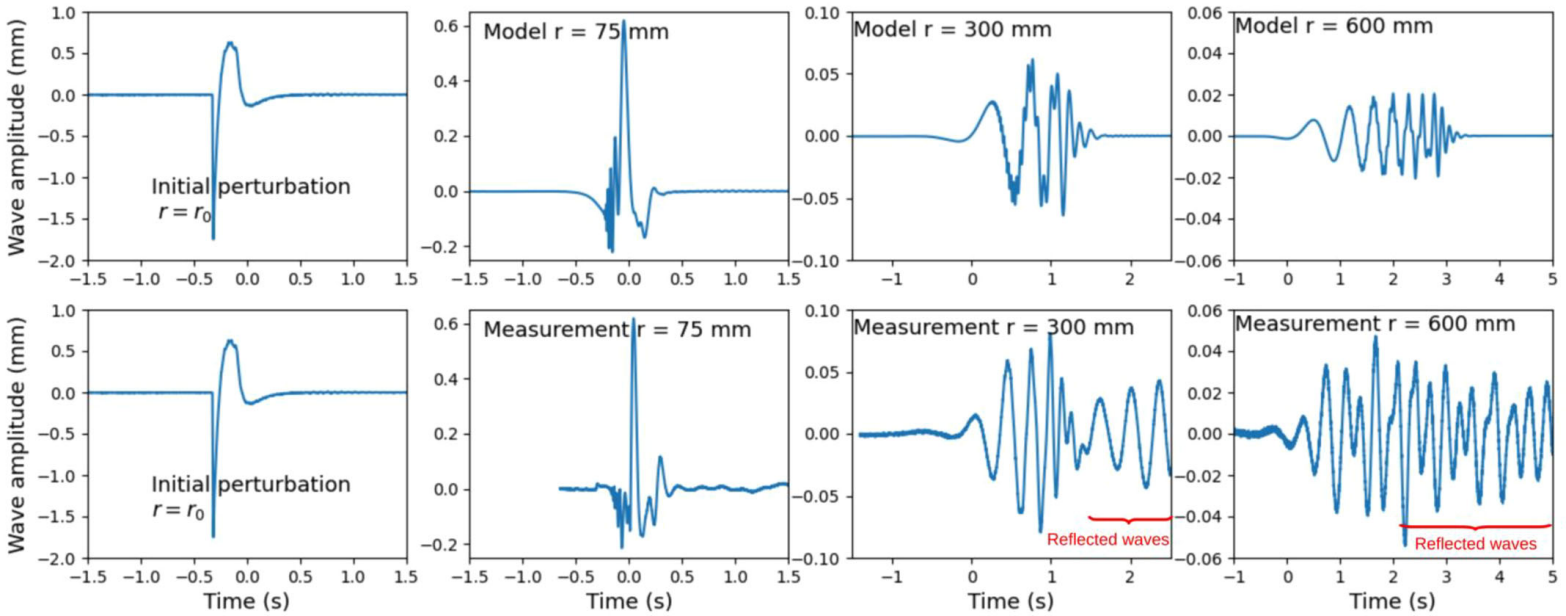
**Model and experimental time signals.** Model signals (on top) are compared to measured signals (at the bottom), for the same distances *r* from the centre of the striking disk. The water depth considered for the calculation is *H*=22 cm, the disk radius is *r*_0_=25 mm and the physical properties of water are *ρ* =998 kg m^−3^, *γ* =72.8×10^−3^ Nm^−1^, *g*=9.81 m s^−2^ and *ν* =1.01×10^−6^m^2^ s^−1^.

The propagation of waves at water surface is subject to dispersion. In other words, waves of different wavelengths *λ* (or frequencies) travel at different speeds. The water surface wave stimulus used in this study was produced by a circular disk striking the water surface. This initial perturbation has the shape of a single pulse followed by a rebound (Fig. 5). Such a time signal is not characterized by a single wavelength but it can be decomposed into a large spectrum of sinusoidal waves by means of a Fourier Transform. Each component of this spectrum travelling at different velocity, the shape of the perturbation changes while it propagates, leading to elongated signals from left to right (Fig. 5), for increasing distances.

This behaviour can be described by considering elementary circular waves originating from the centre of the disk, with complex representation of the form:

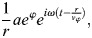
where *ω* is the wave pulsation and 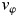 its phase velocity.

Considering a finite water depth H, the dispersion relation of wave at the water surface is written as (Guyon et al., 2015):
(1)

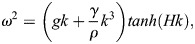
where 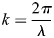 is the wave number, *g* the gravity constant and *ρ* and *γ* the density and surface tension of water. Introducing the definition of the phase velocity 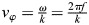 and the wave frequency *f*

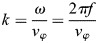
Eqn (1) leads to:
(2)

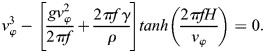


**Fig. 6.**
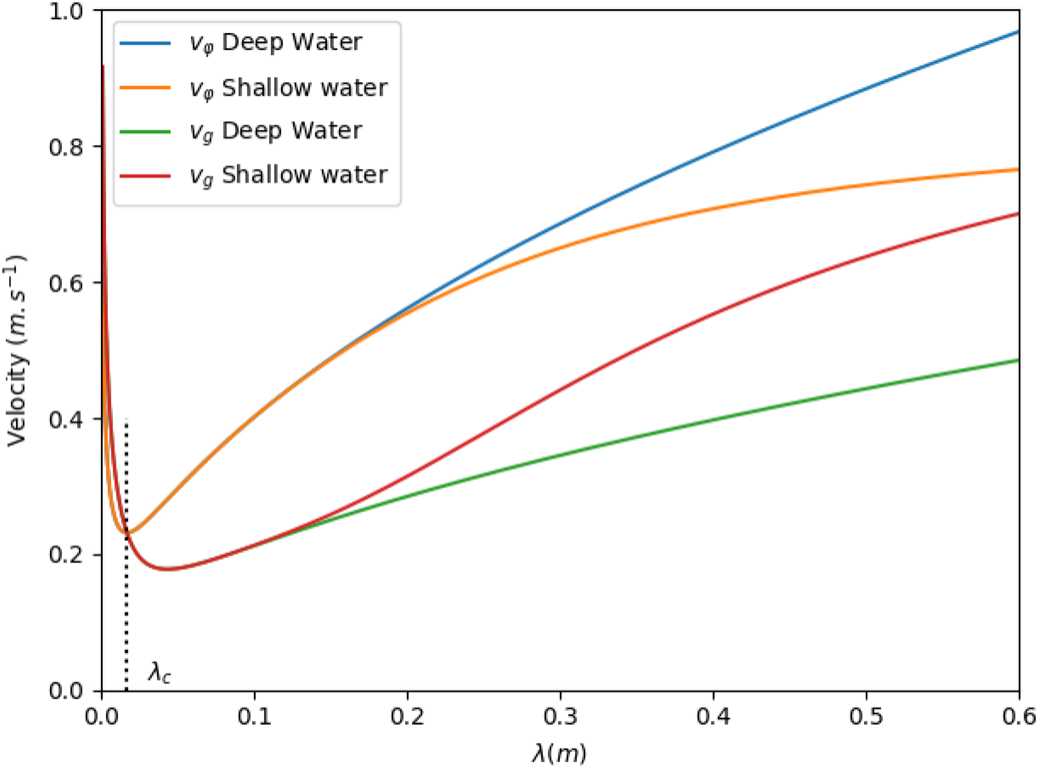
**Surface waves dispersion.** Phase and group velocities as a function of wavelength *λ*, with and without deep water approximation. The phase velocity strongly depends on the wavelength. As a consequence, the different components of a complex waveform propagate at different velocities, leading to constant deformation of this waveform. The water depth in shallow water case is *H*=7 cm. The physical properties used for these calculations are for water at 20° and atmospheric pressure: *ρ* =998 kg m^−3^, *γ* =72.8×10^−3^Nm^−1^, and *g*=9.81 m s^−2^.

This equation can be solved numerically to evaluate 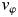 as a function of *f* or *λ* as shown in Fig. 6. The phase velocity is the velocity of a single sinusoidal wave of wavelength *λ*, i.e. a single component of the spectrum. It differs from the group velocity *v*_*g*_, which is the velocity of the envelop of a wave packet whose central wavelength is *λ*, which satisfies:

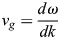

(3)

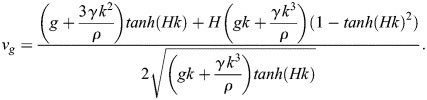


In deep water, typically for water depth *H* greater than *λ*/2, the hyperbolic tangent terms in Eqns 1, 2 and 3 reduce to 1 and this set of equations is greatly simplified.


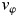
 are represented in Fig. 6 as a function of *λ* in deep water approximation and for shallow water with *H*=7 cm, the minimum water depth considered in this study. For this minimum value, the phase and the group velocities depart significantly from the velocities calculated with deep water approximation for wavelength greater than 0.15 m. For greater *H*, the departure is visible for *λ*>2*H*. In any case, the phase velocity decreases for *λ* smaller than the capillary wavelength *λ*_*c*_ and increases for *λ*>*λ*_*c*_. The capillary wavelength 
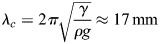
 corresponds to the lower value of the phase velocity. It also marks the limit between capillary waves (for *λ*<*λ*_*c*_) and gravity waves for (*λ*>*λ*_*c*_). In the present study, the two kinds of waves must be considered to describe the wide spectrum characterizing the initial perturbation.

While they propagate, the surface waves are attenuated by the effect of viscosity *ν*. In deep water and clean surface conditions, the amplitude decreasing in space due to viscosity can be written as (Phillips, 1977):
(4)

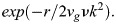
The wave propagation can be simulated using this set of equations (1-4), with and without deep water approximation, to describe at least qualitatively, the waves' behaviours in our experiments. The displacement of the striking disk is measured versus time, in air (Fig. 5). This time signal is considered as the initial circular perturbation of the water surface, at the edge of the disk, i.e. at distance *r*=*r*_0_=25 mm from the centre, and time *t*=0.

The time signal of the perturbation is decomposed into a spectrum of elementary waves by means of a Fourier transform. The propagation of each elementary wave over a distance is considered individually. It is described by a phase shift and a decay of amplitude. Each component of the Fourier transform of frequency *f* is then simply multiply by:

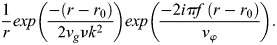


Finally, the time signal after propagation, at distance *r* is obtained by calculating the inverse Fourier transform of the modified spectrum.

The initial perturbation consists of a main peak followed by a rebound as shown in Fig. 5 in which model time signals, calculated at different propagation distances *r* are shown at the top and experimental signals for the same distances *r* at the bottom. Experimental and model signals show similar trends, despite the strong assumptions made for the calculations (single point perturbation at centre of the striking disk, one-dimensional wave, clean water). Along the first tens of millimetres, the shape of the disturbance changes continuously and very quickly. At *r*=75 mm (50 mm from the disk boundary), both model and experimental signals show some high frequency oscillations, followed by a high peak and rebounds. The very high frequency components (low wavelengths) arrive first as they are associated to the highest phase velocities (see Fig. 6 for *λ*<*λ*_*c*_). At *r*=300 mm, the highest frequencies have almost vanished, due to the viscous attenuation (see Eqn 4), stronger for larger frequencies (lower wavelengths). Among the remaining components, the lower frequencies, with higher phase velocities, arrive first. The resulting time signal is a chirped signal, with an instantaneous frequency increasing in time. At *r*=600 mm, the signal duration has elongated, it is still chirped with an increasing frequency versus time. The main difference between model and experimental signals are due to the presence of reflected waves at the wall of the tank used in the laboratory experiments. These reflected waves are delayed with respect to the main signal, due to a longer travel path. Their arrival time can be estimated from the tank geometry and the group velocity (about 1.5 s at 300 mm and 2.0 s at 600 mm) and excluded from the signal analysis during the calibration procedure. The presence of reflections limits the distance at which the calibration measurements can be done. From a quantitative point of view, the attenuation of the wave with propagation distance is not well reproduced by the model.

Similar calculations assuming the deep-water approximation (not presented) show similar trends. The time signal changes with water depth for smaller than 150 mm, as the phase velocity of the largest wavelength (lower frequency) components changes, as illustrated below. However, the amplitude of the oscillations, i.e. the maximum peak to peak variation of these oscillations, as measured in the calibration procedure remains unchanged for the water depth considered in this study (*H*>7 cm).

From now, the surface waves have been described at single point (fixed distance *r*) as a function of time *t*, to be directly compared to the measurements. An alternative and probably more intuitive and meaningful way to represent the wave propagation consists in plotting the water height along a line, as a function of *r*, at given time *t*. Time *t* is then increased to build the joined video (see data availability and resource section). The video illustrates the modification of the wave form during propagation. It shows how lower frequency components propagates faster and arrive first at a given distance. It also shows the weak influence of deep-water approximation. Considering the distances between wave stimuli and crocodiles in the present study, an important conclusion is that the shape, the duration and the amplitude of the surface waves as received by the crocodile are completely different from those of the initial perturbation. Since it is very difficult to measure these very low-amplitude waves at great distances, the use of a propagation model and calibration procedure at shorter distances offers an interesting alternative for describing the waves as perceived by the crocodiles.

## Supplementary Material

10.1242/biolopen.061844_sup1 Supplementary information
