# Supplementary material for: Temporal integration and decision-making in crocodiles
Source: Biol Open. 2025 May 6;14(5):bio061844. doi: 10.1242/bio.061844 (PMC12079573; doi:10.1242/bio.061844)
Supplement: Supplementary information [file biolopen-14-061844-s1.pdf]

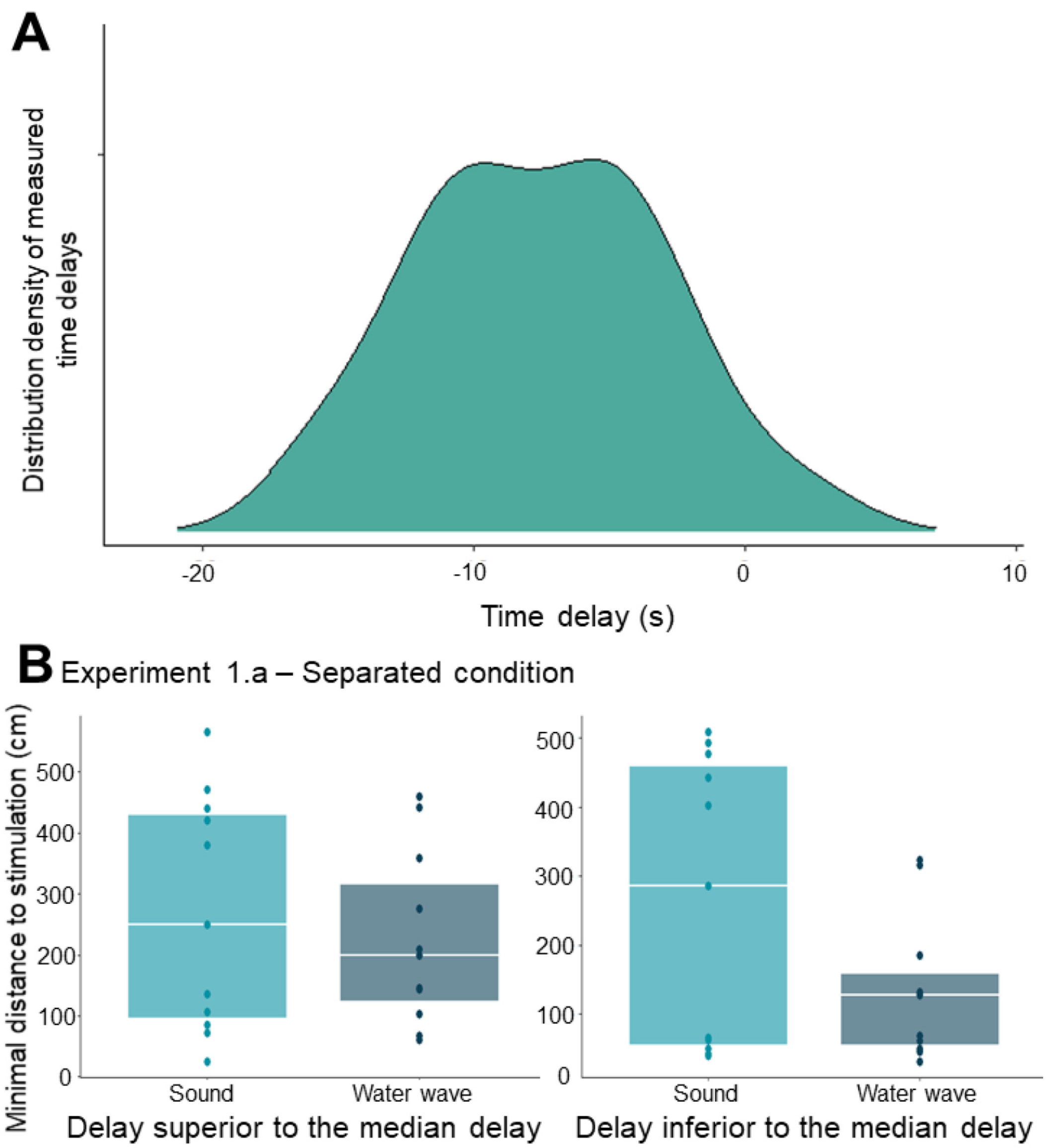

Fig. S1. Effect of the delay between the two stimuli on the behavioral response of crocodiles. (A) Distribution of the duration of the delays between the arrival of water surface waves and sound waves at the crocodile (density is unitless, representing the probability distribution of the data). The delays were measured from the videos, as a function of the crocodile's position relative to the vibration and sound sources. (B) The preference to approach the vibration source was observed for delays shorter than the median delay. This preference was not visible for longer delays in the experiment 1.a. Boxplots represent the minimum distance to the stimulus source reached by the crocodile.

Table S1. Comparison of approach distances between the sound emitted by the disc of the vibrating device ("DVD") and the experimental stimuli ("Test"). "Fit": values fitted by the Bayesian model; "Se": standard error; "Lwr": value below 2.5% of the fitted values lie; "Upr": value above 2.5% of the fitted values.

Variable	Stimulus	Fit	Se	Lwr	Upr
Minimal distance (cm)	DVD	169	1	130	232
	Test	119	1	100	141

Table S2. Fitted values of minimal distance (cm) to stimulus source for experiment 1. Fit represents the median fitted minimal distance for all iterations of the bayesian model, Se the standard error, Lwr the value below which 2.5% of the fitted values are and Upr the value above which 2.5% of the fitted values are.

Experiment	Stimulus	Fit	Se	Lwr	Upr
Experiment 1.a	Sound	185	1	124	266
	Water Wave	134	1	89	194
Experiment 1.b	Sound	252	1	188	341
	Water Wave	211	1	159	279
	Sound + Water Wave	243	1	182	327

Biology Open • Supplementary information

Table S3. Fitted values of minimal distance (cm) to sound and water wave source for all temporal conditions in experiment 2. The values for “All conditions” represent the results for a model runned with the first three temporal conditions (Sound - Water Wave, synchronized and Water Wave - Sound). Fit represents the median fitted minimal distance for all iterations of the bayesian model, Se the standard error, Lwr the value below which 2.5% of the fitted values are and Upr the value above which 2.5% of the fitted values are.

Temporal condition	Stimulus	Fit	Se	Lwr	Upr
Sound - Water Wave	Sound	201	1	161	249
	Water Wave	147	1	118	181
Synchronized	Sound	167	1	126	226
	Water Wave	122	1	92	158
Water Wave - Sound	Sound	227	1	182	281
	Water Wave	147	1	119	180
Natural	Sound	136	1	102	180
	Water Wave	141	1	106	185
All conditions	Sound	191	1	166	218
	Water Wave	139	1	121	159

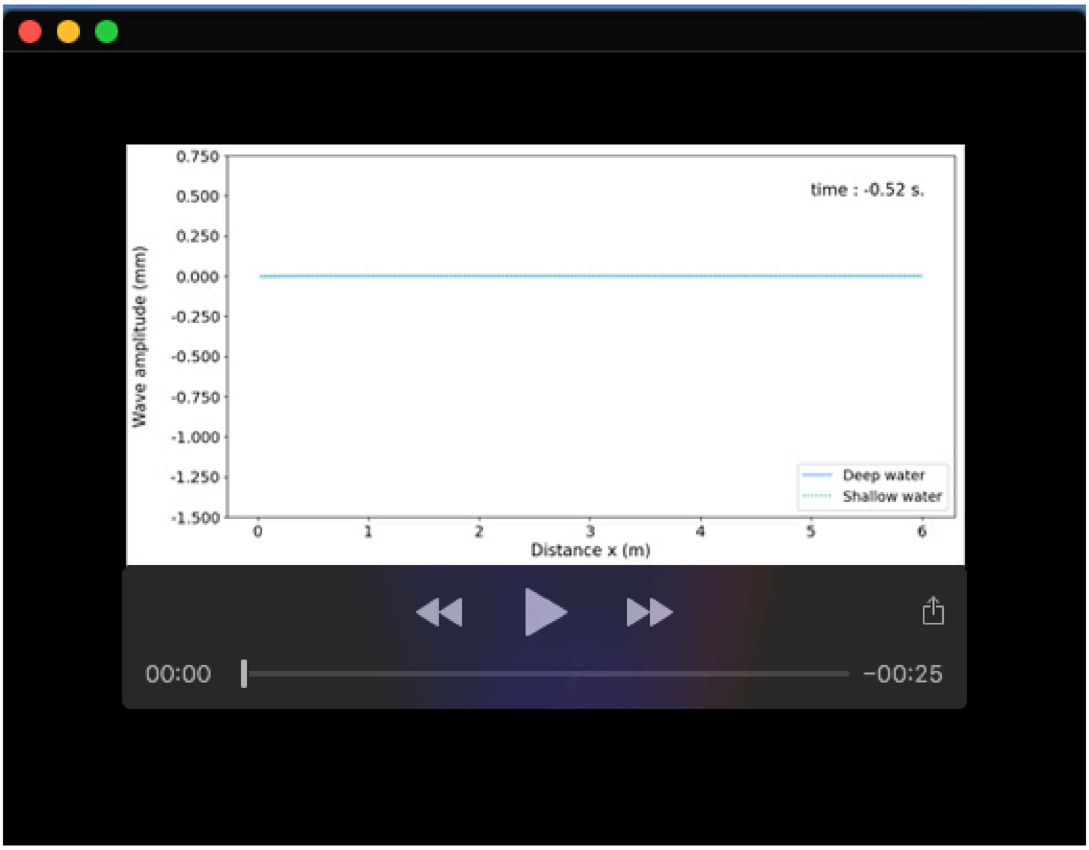

Movie 1. Modelling propagation of water surface waves.

The animate shows the evolution in time of the water height along a radial line. The water height profiles are calculated following Eqn. (1) to (4), in deep water and shallow water conditions. The animate illustrated how the initial perturbation changes during propagation, how it elongates in time and how the lowest frequency travel faster than the others. The vertical scale changes continuously during the video to compensate the fast decay of the waves' amplitudes.

Biology Open • Supplementary information
